# Arterial stiffness is associated with handgrip strength in relatively healthy Chinese older adults

**DOI:** 10.3389/fnut.2024.1342411

**Published:** 2024-02-09

**Authors:** Yan He, Yue Niu, Zhe Li, Ruimin Zhang, Yizhi Chen, Zheyi Dong, Ying Zheng, Qian Wang, Yong Wang, Delong Zhao, Xuefeng Sun, Guangyan Cai, Zhe Feng, Weiguang Zhang, Xiangmei Chen

**Affiliations:** ^1^Department of Nephrology, First Medical Center of Chinese PLA General Hospital, National Key Laboratory of Kidney Diseases, National Clinical Research Center for Kidney Diseases, Beijing Key Laboratory of Kidney Diseases Research, Beijing, China; ^2^Chengdu University of Traditional Chinese Medicine, Chengdu, China; ^3^The First Affiliated Hospital and College of Clinical Medicine of Henan University of Science and Technology, Luoyang, China; ^4^Department of Nephrology, Hainan Hospital of Chinese PLA General Hospital, Hainan Province Academician Team Innovation Center, Sanya, China

**Keywords:** handgrip strength, brachial-ankle pulse wave velocity, arterial stiffness, older adults, healthy

## Abstract

**Background:**

Increased arterial stiffness and low handgrip strength (HGS) are associated with poor health outcomes and are a severe health risk for older adults. However, there is limited evidence and mixed results on whether there is an association between them. Therefore, this study focused on the association between arterial stiffness and HGS in relatively healthy older adults in Beijing, China.

**Methods:**

In 2016, 2,217 adult volunteers were recruited in Beijing. Brachial-ankle pulse wave velocity (baPWV) and the ankle-brachial index were measured using an automatic vascular profiling system. Carotid artery intima-media thickness and common carotid artery-internal diameter (CCAID) were evaluated using Doppler ultrasound, and HGS was measured with a dynamometer. Low HGS was determined using the Asian Sarcopenia Working Group 2019 criteria. Multivariate linear and logistic regressions evaluated the relationship between arterial stiffness and HGS.

**Results:**

Ultimately, 776 relatively healthy older adults (mean age 69.05 ± 6.46 years) were included. Based on the AWGS2019 criteria, 137 participants were defined as having low HGS. Compared to the normal HGS group, the low HGS group was older and had higher baPWV (*p* < 0.001) but lower CCAID, body mass index (BMI) and hemoglobin (Hb) (*p* < 0.05). The multiple linear regression analysis revealed that baPWV was negatively correlated with HGS (*β* = −0.173, *t* = −2.587, *p* = 0.01). Multivariate logistic regression analysis showed that baPWV and CCAID were associated with an increased risk of low HGS (odds ratio (OR) per SD increase: 1.318, *p* = 0.007; OR per SD increase: 0.541, *p* < 0.001).

**Conclusion:**

Arterial stiffness and HGS were significantly negatively correlated in relatively healthy Chinese older adults. Low HGS is associated with increased arterial stiffness. Encouraging exercise training to improve HGS, thereby reducing arterial stiffness and the risk of cardiovascular events, may be a simple and effective intervention.

## 1 Introduction

Handgrip strength (HGS) is often used in clinical practice because it is simple and noninvasive (1). HGS is an important indicator for diagnosing sarcopenia and can assess the body’s overall muscle strength (1). Previous studies have suggested that the role of low muscle strength may outweigh the role of low muscle mass and thus become the main determinant of the diagnosis of sarcopenia (2). HGS can also objectively and accurately reflect the body’s nutritional status and is reliable for predicting malnutrition (3, 4). In addition, HGS may also be a predictor of arterial stiffness in identifying the risk of cardiovascular disease and mortality (5, 6). It is also associated with adverse health outcomes such as fractures, hospitalization rates, cardiovascular events, and all-cause mortality, which seriously harm individual health (1, 4, 7). Arterial stiffness is one of the earliest indicators of structural and functional changes in the vascular wall and is associated with diminished arterial elasticity (8). The more pronounced the arterial stiffness is, the greater the risk of cardiovascular disease (9). There are various noninvasive methods for evaluating arterial stiffness, such as carotid-femoral pulse wave velocity (cfPWV) and brachial-ankle pulse wave velocity (baPWV). All of these methods involve calculations of the speed of pulse waves along the artery tree. Both evaluate the pulse wave velocity of the aorta, but the measurements are different. The cfPWV evaluates the pulse-wave velocity from the carotid artery to the femoral artery, while the baPWV evaluates the pulse-wave velocity from the brachial artery to the ankle artery (10). The cfPWV is the gold standard for assessing central arterial stiffness (10). However, baPWV is also widely used in many studies, and baPWV is strongly correlated with cfPWV and is a recognized marker of arterial stiffness (11–13). In addition, the ankle-brachial index (ABI), as a surrogate for arterial stiffness, is associated with the risk of cardiovascular events (14). Carotid artery intima-media thickness (CIMT) is an excellent surrogate marker of coronary atherosclerosis (15, 16). In addition, the common artery internal diameter (CCAID) was negatively correlated with the maximum CIMT, which is associated with carotid atherosclerosis, a predictor of cardiovascular disease (16–18). Like low HGS, arterial stiffness is associated with cardiovascular event occurrence and all-cause mortality (16, 19). There is a complex relationship between HGS and arterial stiffness. Some theories suggest that arterial stiffness develops after molecular and cellular inflammation (20). At the same time, inflammatory factors such as tumor necrosis factor and interleukin 6 are essential factors that inhibit muscle synthesis and promote muscle atrophy and are negatively correlated with muscle strength (21, 22). Meanwhile, muscle cell atrophy also affects the oxidation state of the body to a certain extent, which can lead to chronic inflammation and vascular sclerosis. Vascular sclerosis leads to endothelial dysfunction, leading to dysregulation of blood flow, reduced microcirculation, and hypoperfusion of muscle tissue, possibly resulting in decreased muscle strength, muscle mass loss, and muscle atrophy (13). Therefore, we hypothesized an association between arterial stiffness and HGS.

Previous studies have also confirmed the relationship between arterial stiffness and HGS. HGS decreased progressively with increasing levels of baPWV in older adults without significant cardiovascular disease, and lower HGS was shown to be associated with increased arterial stiffness (23). A cohort study of aging confirmed that arterial stiffness might contribute to lower HGS and is a risk factor for low HGS (24). In contrast, a cohort study with a two-year follow-up noted that HGS was not associated with arterial stiffness (25). A recent study demonstrated that higher muscle strength was associated with lower CIMT (26). When CIMT was used as an indicator of arterial stiffness, arterial stiffness was associated with HGS levels, even after adjusting for confounding factors such as sex and age (27). Using the ABI to assess arterial disease, HGS was shown to be related to peripheral arterial disease in older hospitalized subjects (28). However, another prospective cohort study indicated that HGS was not associated with the ABI and that gait speed was associated with the ABI (29).

As a result, the current evidence on the relationship between HGS and arterial stiffness needs to be more comprehensive, and some results need to be confirmed in a more conclusive manner. Most studies only use one or two arterial stiffness indicators to explore the correlation between the above variables. At the same time, we were unable to find any literature to show whether the change in CCAID is related to low HGS. Arterial stiffness is an important risk factor for cardiovascular disease (13). HGS is a commonly used indicator in clinical practice that has the advantages of being a simple and noninvasive measurement and may be able to predict arterial stiffness (5, 6). Moreover, improving HGS through exercise training is easy and feasible (30, 31). Using a combination of arterial stiffness markers, we conducted this study to establish an association between HGS and arterial stiffness in relatively healthy older people. We propose a clinical method of strengthening HGS through exercise training to improve arterial stiffness and reduce the risk of cardiovascular disease.

## 2 Materials and methods

### 2.1 Study design and participants

This study was conducted at Chinese PLA General Hospital in 2016 and initially recruited 2,217 volunteers aged ≥18 years from Beijing, China, according to the following exclusion criteria: (1) age < 60 years; (2) subjects with respiratory system diseases such as chronic obstructive pulmonary disease, asthma, and bronchiectasis; (3) volunteers with fractures, rheumatoid arthritis and other rheumatic diseases; (4) individuals who had suffered from one of the following diseases: diabetic mellitus, hypertension, chronic kidney disease, cirrhosis, stroke, myocardial infarction, malignant hematologic diseases and malignant tumors; and (5) people who were unwilling to cooperate with indicator testing and missing sample data. Since there is no fixed standard for healthy people, we considered the individuals who were excluded based on those mentioned above clinically common or vital organ diseases “relatively healthy individuals.” Finally, 776 relatively healthy older adults were included in the study (Supplementary Figure S1). This study was conducted in accordance with the Declaration of Helsinki, and the Ethics Committee of Chinese PLA General Hospital approved the research plan. All volunteers signed informed consent forms before participating in this study.

### 2.2 Clinical data collection

Demographic information (sex, age) was collected from participants who wore light clothing and were barefoot. The height and weight of the participants were measured with a stadiometer (Seca 213, Hamburg, Germany) and a digital weighing scale (Omron HN-289-BK, Kyoto, Japan), respectively. Soft, nonstretchable plastic tape was used to measure waist and hip circumferences at the navel level and at the widest point of the hips. In a calm resting state, the participant was in a seated position, and the blood pressure in the dominant arm was measured using the automated electronic device Omron HEM-757 (Omron Healthcare, Kyoto, Japan) three times, with each interval of 1-min rest. The average systolic blood pressure (SBP) and diastolic blood pressure (DBP) were recorded for analysis. BMI was calculated by weight (kg)/height (m^2^), and pulse pressure (PP) was calculated by SBP minus DBP. Fasting venous blood was collected in the morning for biochemical analysis. The automatic biochemical analyzer Roche cobas 8,000, C701 module (Roche Diagnostics, Mannheim, Germany) was used for blood biochemical analysis with Roche’s original reagent and spectrophotometric methods. A complete blood count and hemoglobin analysis were performed by means of an automated hematology analyzer (Sysmex XN-9000, Kobe, Japan). Hemoglobin (Hb), albumin (Alb), total protein (TP), fasting glucose (FPG), triglycerides (TG), total cholesterol (TC), high-density lipoprotein cholesterol (HDL-C), serum creatinine (SCr), urea (Urea) and other indicators were recorded. The estimated glomerular filtration rate (eGFR, calculated according to the Chronic Kidney Disease Epidemiology Collaboration (CKD-EPI) equation based on creatinine) (32).

### 2.3 Measurement of HGS and arterial stiffness

HGS was measured using a Jamar dynamometer (Sammons Preston Rolyan, Bolingbrook, IL, United States). Subjects assumed a seated position. Three maximal-effort isometric contractions were performed in each hand, with a rest of at least 30 s allowed between measurements, and the maximum force values of the left and right hands were recorded. In this study, the average value of the maximum HGS of the two hands was taken for statistical analysis. Subjects were evaluated using a vascular profiling system (Omron VP-1000, Kyoto, Japan), which recorded both baPWV and the ABI simultaneously. In this study, larger baPWV values on both sides were selected for analysis. The ABI is the ratio of the systolic pressure of the ankle artery to that of the brachial artery. In general, the analysis was performed with a lower value of the left and right ABI, but when the higher ABI was greater than 1.3 and the lower ABI was normal (1.0–1.3), ABI > 1.3 was used for the analysis. CIMT is the distance between the epithelial-medial interface and the intimal-luminal interface (16). CCAID is the mean of the minimum distance between the intima-lumen of the proximal and distal walls of the common carotid artery (18). Using Doppler ultrasound (Philips IE33 ultrasound system, The Netherlands), the CIMT and CCAID were measured at a selected location in the middle common carotid artery and analyzed using the mean values of the left and right sides. Medical professionals performed all operations.

### 2.4 Definitions

Low HGS was defined based on The Asian Working Group for Sarcopenia, 2019 (AWGS2019) HGS cutoff values (HGS < 28 kg for males and HGS < 18 kg for females) (1). The Committee for the Physical Diagnosis of Vascular Failure recommends that the cutoff values for the diagnosis of vascular failure based on baPWV are 14 m/s and 18 m/s (<14 m/s is normal, 14–18 m/s is borderline, and ≥ 18 m/s is abnormal) (33). In this study, subjects with baPWV≥18 m/s were judged to have arterial stiffness.

### 2.5 Statistical analysis

Continuous variables are presented as the means ± standard deviations (SD) for normally distributed data or as the medians (first and third quartiles) for skewed data. Categorical variables are presented as counts and percentages (%). The independent t test, the chi-square test or the Mann–Whitney U test was used to compare numerical data between the two groups. The relationship between arterial stiffness and HGS was analyzed by multiple linear regression, and the model was adjusted according to sex, age, PP, GLU, Hb, TP, eGFR and BMI. The multivariate model was established by binary logistic regression, with numerical variables standardized for consistency. The odds ratios (ORs) and 95% confidence intervals (95% CIs) between the arterial stiffness index and low HGS were analyzed by forward stepwise regression. All data were analyzed by the statistical software SPSS26.0 for Mac (SPSS, Chicago, IL, United States) and R 4.3.1 via the packages mice version 3.16.0, and package forestmodel version 0.6.2. Differences were considered statistically significant at *p* values of <0.05.

## 3 Results

### 3.1 Characteristics of participants

Table 1 lists the clinical characteristics of participants with low HGS and normal HGS. A total of 776 older adults were included, including 436 females (56.2%), with a mean age of 69.05 ± 6.46 years. The mean baPWV was 16.59 ± 3.26 m/s (30.2% baPWV≥18 m/s), the mean HGS was 27.22 ± 9.06 and the mean CCAID was 6.87 ± 1.4 mm. According to the criteria of AWGS2019, 137 participants (17.7%) were diagnosed in the low HGS group. Compared with the normal HGS group, the low HGS group was older and had higher baPWV and PP (*p* < 0.05) and lower BMI, CCAID, Hb, height, weight and eGFR (*p* < 0.05), but there were no differences observed for CIMT or the ABI (*p* > 0.05).

**Table 1:** Comparison of clinical characteristics between normal HGS and low HGS participants.

Variables	Normal HGS	Low HGS	Overall	Value of *p*
*N* (%)	639	137	776	
Sex				0.036 ^*^
Male, *n* (%)	291 (45.5)	49 (35.8)	340 (43.8)	
Female, *n* (%)	348 (54.5)	88 (64.2)	436 (56.2)	
Age (years)	68.10 ± 5.92	73.44 ± 7.05	69.05 ± 6.46	<0.001^**^
Height (cm)	164.36 ± 7.72	159.61 ± 7.56	163.52 ± 7.9	<0.001^**^
Weight (kg)	66.49 ± 10.17	60.06 ± 9.49	65.36 ± 10.34	<0.001^**^
WC (cm)	88.33 ± 9.18	86.71 ± 9.41	88.04 ± 9.23	0.062^**^
HC (cm)	99.18 ± 6.26	97.31 ± 6.94	98.85 ± 6.42	0.002^**^
BMI (kg/m^2^)	24.57 ± 3.12	23.55 ± 3.18	24.39 ± 3.15	0.001^**^
SBP (mmHg)	129.50 ± 15.64	132.35 ± 16.85	130.00 ± 15.89	0.056^**^
DBP (mmHg)	73.93 ± 9.23	72.60 ± 9.83	73.69 ± 9.35	0.131^**^
PP (mmHg)	55.51 ± 10.92	59.75 ± 11.86	56.26 ± 11.21	<0.001^**^
HGS (kg)	29.31 ± 8.29	17.51 ± 5.48	27.22 ± 9.06	<0.001^**^
BaPWV(m/s)	16.26 ± 3.09	18.08 ± 3.63	16.59 ± 3.26	<0.001^**^
<18 m/s, n (%)	476(84.8)	66(48.2)	542(69.8)	
≥18 m/s, n (%)	163(15.2)	71(51.8)	234(30.2)	
CIMT (mm)	1.00 ± 0.37	0.98 ± 0.22	1.00 ± 0.35	0.573^**^
CCAID (mm)	6.95 ± 1.46	6.52 ± 1.02	6.87 ± 1.40	0.001^**^
ABI	1.11(1.06, 1.15)	1.11(1.06, 1.14)	1.11(1.06, 1.15)	0.740^***^
Hb (g/L)	141.93 ± 12.77	135.82 ± 13.21	140.85 ± 13.05	<0.001^**^
ALB (g/L)	45.77 ± 2.85	45.26 ± 2.51	45.68 ± 2.80	0.052^**^
TP (g/L)	74.80 ± 4.47	74.72 ± 4.36	74.79 ± 4.45	0.850^**^
FPG (mmol/L)	5.31(4.89, 5.92)	5.32(4.83, 5.92)	5.32(4.89, 5.92)	0.690^***^
TG (mmol/L)	1.33(0.96, 1.81)	1.2(0.85, 1.74)	1.31(0.94, 1.8)	0.074^***^
TC (mmol/L)	4.78 ± 0.93	4.72 ± 1.13	4.77 ± 0.97	0.624^**^
HDL-C (mmol/L)	1.43 ± 0.37	1.55 ± 0.38	1.45 ± 0.37	0.001^**^
SCr (umol/l)	75.92 ± 15.35	77.65 ± 23.01	76.22 ± 16.95	0.400^**^
BUN (mmol/l)	5.37 ± 1.30	5.77 ± 1.68	5.44 ± 1.38	0.009^**^
eGFR (ml/min × 1.73 m^2^)	80.01 ± 12.42	75.01 ± 15.18	79.13 ± 13.08	<0.001^**^

Categorical variables are expressed as numbers (percentages) and continuous variables are expressed as mean standard deviation or median (medians (first and third quartiles)). ^*^Chi-square test; ^**^Student’s *t*-test; ^***^Mann–Whitney U test. WC, Waist circumference; HC, Hip circumference; BMI, body mass index; SBP, systolic blood pressure; DBP, diastolic blood pressure; PP, pulse pressure; HGS, handgrip strength; BaPWV, brachial-ankle pulse wave velocity; CIMT, carotid intima–media thickness; CCAID, common carotid artery internal diameter; ABI, ankle-brachial index; Hb, hemoglobin; ALB, albumin; TP, total protein; FPG, fasting plasma glucose; TG, triglycerides; TC, total cholesterol; HDL-C, high density lipoprotein cholesterol; SCr, serum creatinine; BUN, blood urea nitrogen; eGFR, estimated glomerular filtration rate.

### 3.2 Characteristics of HGS in different baPWV groups

**Figure 1:**
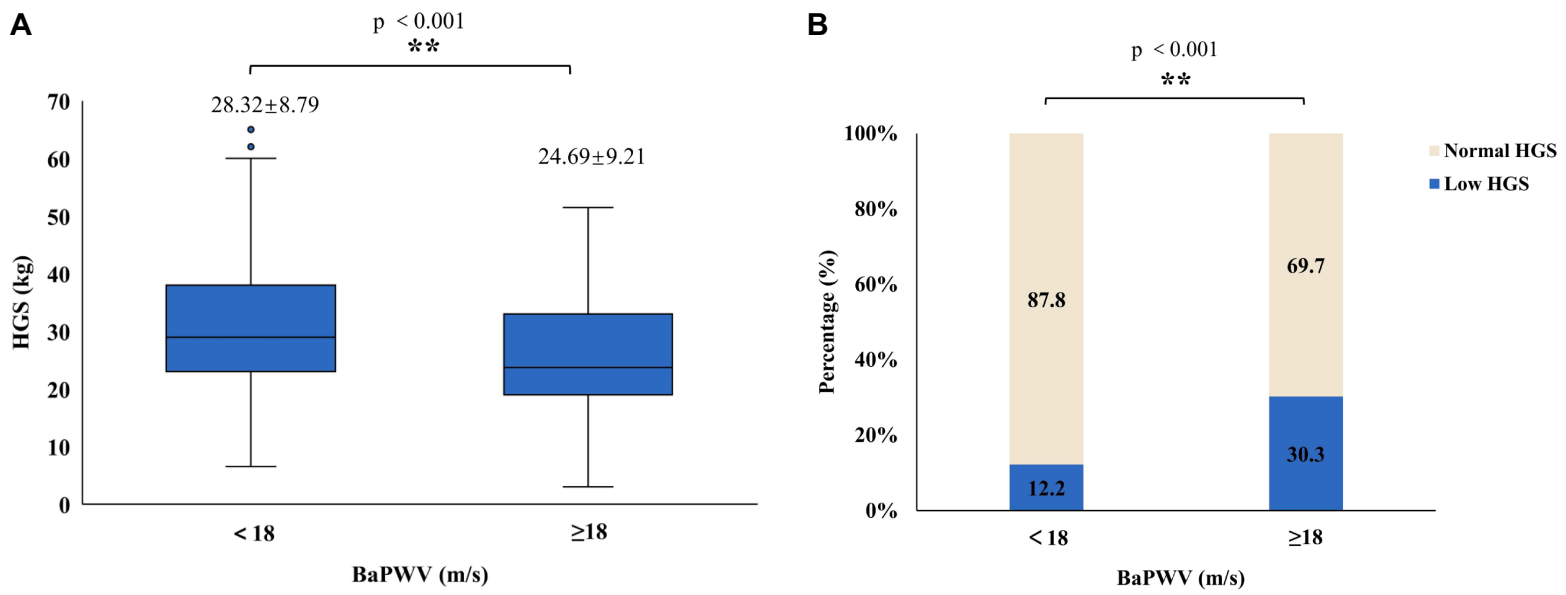
Characteristics of HGS in different baPWV groups: **(A)** boxplots showing distributions of HGS between the different baPWV groups; **(B)** stacked bar plots of low HGS percentage (navy blue bar) and normal HGS percentage (ivory white bar) between different baPWV groups. HGS, handgrip strength; baPWV, brachial-ankle pulse wave velocity.

Participants were divided into two groups (baPWV≥18 m/s and baPWV<18 m/s) with baPWV at 18 m/s as the cutoff value for arterial stiffness to explore the characteristics of HGS in the two groups, as shown in Figures 1A,B. Participants in the baPWV≥18 m/s group had lower HGS (24.69 ± 9.21 kg vs. 28.32 ± 8.79 kg, *p* < 0.001) and a higher proportion of low HGS (30.3% vs. 12.2%, *p* < 0.001). In the arterial stiffness group, the HGS was low, and the proportion of low HGS was high.

### 3.3 Multiple regression analysis for HGS

To understand the relationship between arterial stiffness and HGS, a multiple linear regression model (Table 2) was established with HGS as the dependent variable and baPWV, CCAID, CIMT and the ABI as the independent variables. The models adjusting for the covariates of sex, age, PP, FPG, Hb, TP, eGFR and BMI were added, combined with collinearity diagnosis, using stepwise regression, and the results showed that with an increase of 1 m/s in baPWV, HGS decreased by 0.173 kg (*t* = −2.587, *p* = 0.010). The decrease in HGS associated with a one-year increase in age was 0.262 kg (*t* = −7.491, *p* < 0.001). BaPWV and age were negatively correlated with HGS.

**Table 2:** Multivariable linear regression analysis of HGS.

Variables	*β*	*t*	Value of *p*
Age (years)	−0.262	−7.491	<0.001
Sex (male,1; female,2)	−12.398	−26.045	<0.001
Hb(g/L)	0.079	4.199	<0.001
BMI (kg/m^2^)	0.178	2.757	0.006
baPWV(m/s)	−0.173	−2.587	0.010

HGS as the dependent variable, baPWV, CCAID, CIMT and ABI as the independent variables. The covariate sex, age, PP, FPG, Hb, TP, eGFR and BMI adjustment models were added. *β* = Coefficient for linear regression; HGS, handgrip strength; baPWV, brachial-ankle pulse wave velocity; CIMT, carotid intima–media thickness; CCAID, common carotid artery internal diameter; ABI, ankle-brachial index; PP, pulse pressure; FPG, fasting plasma glucose; Hb, hemoglobin; TP, total protein; eGFR, estimated glomerular filtration rate; BMI, body mass index.

### 3.4 Multiple logistic regression analysis for low HGS

**Figure 2:**
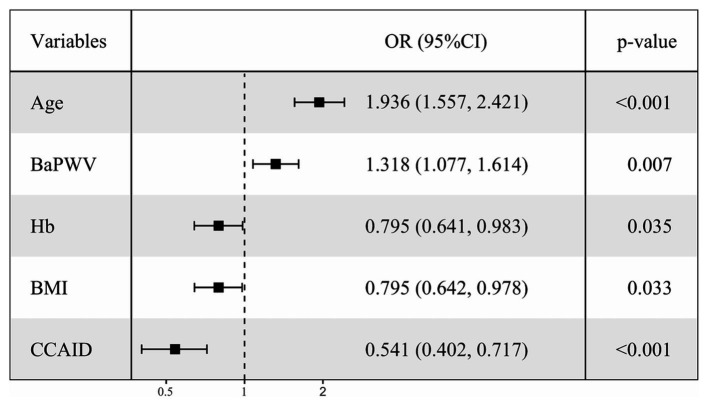
Multiple-adjusted standardized logistic regression analysis of low HGS Low/normal HGS as the dependent variable, with numerical variables standardized for consistency. OR, odds ratio; CI, confidence interval; HGS, handgrip strength; Hb, hemoglobin; BMI, body mass index; baPWV, brachial-ankle pulse wave velocity; CCAID, common carotid artery internal diameter.

**Figure 3:**
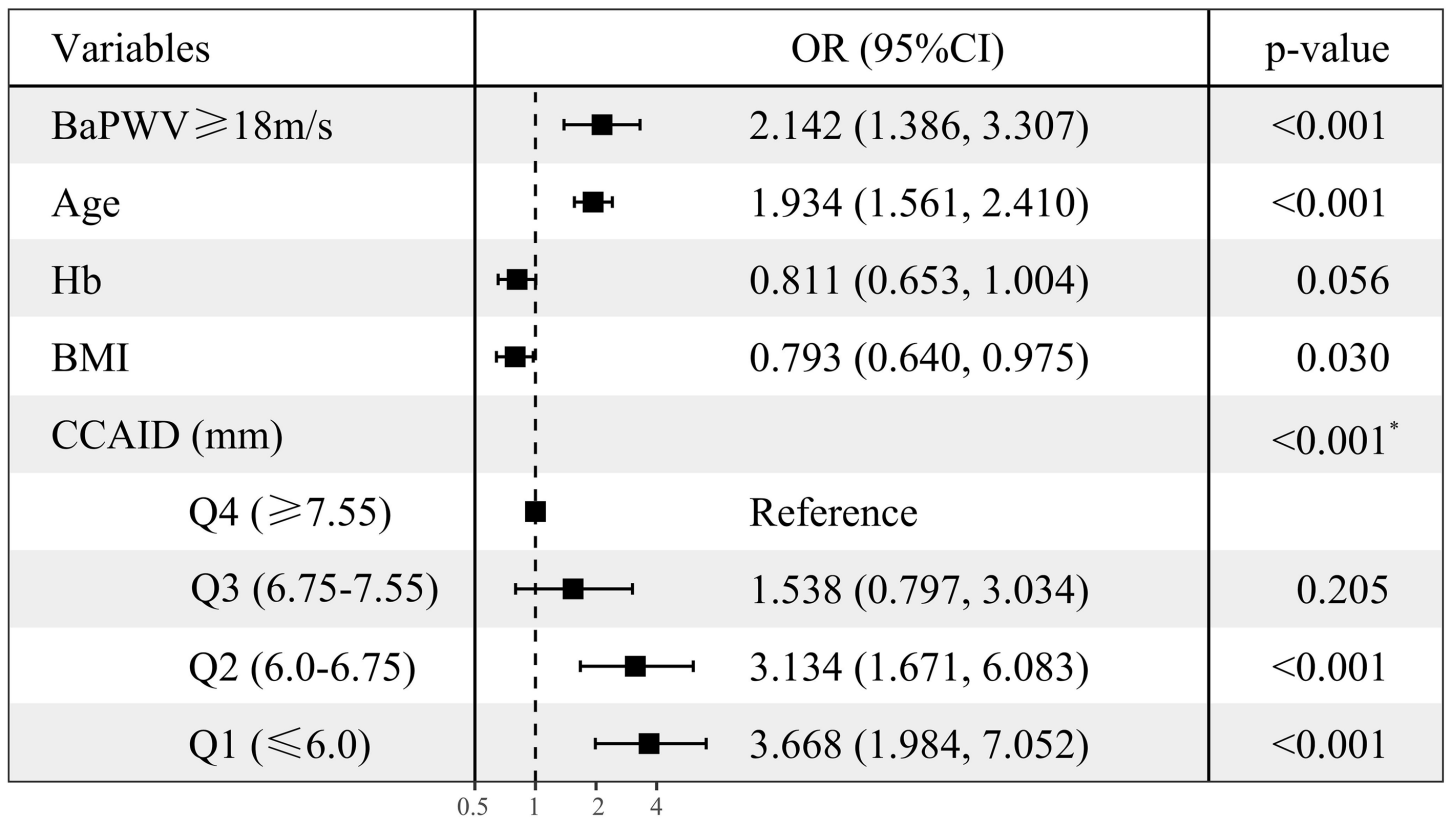
Multiple-adjusted standardized logistic regression analysis of low HGS (Set dummy variables for baPWV, CCAID). Low/normal HGS as the dependent variable, with numerical variables standardized for consistency. OR, odds ratio; CI, confidence interval; HGS, handgrip strength; Hb, hemoglobin; BMI, body mass index; baPWV, brachial ankle pulse wave velocity; CCAID, common carotid artery internal diameter; Q, quartile. ^*^p for trend.

To clarify the factors influencing low HGS, logistic regressions were performed with low/normal HGS as the dependent variable, with numerical variables standardized for consistency. Variables with *p* < 0.1 in univariate logistic regression (Supplementary Figure S2) were selected for multivariate logistic regression analysis (Figure 2), combined with collinearity diagnosis. Finally, age, baPWV, CCAID, SBP, PP, Hb, BMI and eGFR were included in the analysis, and sex was included to control for confounding factors. The results showed that baPWV (OR per SD increase: 1.318; 95% CI: 1.077–1.614; *p* = 0.007) and age (OR per SD increase: 1.936; 95% CI: 1.557–2.421; *p* < 0.001) were the factors that increased the risk of low HGS. Hb (OR per SD increase: 0.795; 95% CI: 0.641–0.983; *p* = 0.035), BMI (OR per SD increase: 0.795; 95% CI: 0.642–0.978; *p* = 0.033). CCAID (OR per SD increase: 0.541; 95% CI: 0.402–0.717; *p* < 0.001) was a risk factor for low HGS, while the ABI, IMT and low HGS were not statistically significant factors (*p* > 0.05) and thus were not included in the analysis. To further explore the relationship between arterial stiffness and low HGS (Figure 3), baPWV was divided into baPWV≥18 m/s and baPWV<18 m/s and CCAID was divided into quartiles (Q1, ≤6.0 mm; Q2, 6.0–6.75 mm; Q3, 6.75–7.55 mm; Q4, ≥7.55 mm); both were corrected for the same variables as above. The risk of low HGS was shown to be 2.142 times higher for baPWV ≥18 m/s than for baPWV <18 m/s (95% CI: 1.386–3.307; *p* < 0.001). Q4 was used as a reference for CCAID, and it was observed that the narrower the CCAID was, the greater the risk of low HGS (Q3: OR = 1.538, 95% CI: 0.797–3.034, *p* = 0.205; Q2: OR = 3.134, 95% CI: 1.671–6.083, *p* < 0.001; Q1: OR = 3.668, 95% CI: 1.984–7.052, *p* < 0.001; *p* for trend<0.001). Thus, advanced age, lower BMI, lower Hb, higher baPWV, and narrower CCAID were associated with an increased risk of low HGS.

## 4 Discussion

Arterial stiffness and low HGS are seriously harmful to individual health. Previous studies have revealed a complex relationship between arterial stiffness and HGS, but the current evidence is limited, and the results are mixed. This study is the first to investigate the association between arterial stiffness and HGS in relatively healthy older people in China by combining multiple arterial stiffness indicators. The results showed that arterial stiffness was significantly negatively correlated with HGS. Higher baPWV and narrower CCAID were associated with an increased risk of low HGS, and for the first time, an association between CCAID and low HGS was observed.

### 4.1 Association between arterial stiffness and HGS

In this study, the baPWV≥18 m/s group had significantly lower HGS and a higher prevalence of low HGS than the baPWV<18 m/s group (*p* < 0.001), and baPWV was significantly negatively correlated with HGS. Previous studies have also reported the relationship between arterial stiffness and HGS. After adjusting for confounding factors such as sex, age, and SBP, the Wakayama Study revealed that HGS decreased with increasing baPWV, and the decrease in muscle strength may be related to the increase in arterial stiffness (23). An Indian and Japanese study observed that a decrease in HGS was significantly associated with an increase in baPWV and CIMT, mainly in nonhypertensive participants; however, in hypertensive participants, arterial stiffness was not associated with HGS, demonstrating an association between arterial stiffness and HGS in nonhypertensive individuals (34). A Brazilian study showed that higher HGS was associated with lower CIMT in adults with cardiovascular disease (*p* < 0.05) (26). Zhang et al. (35) reported that after adjusting for confounding factors such as sex, age, blood pressure and heart rate, HGS decreased by 0.13 kg per 1-SD increase in baPWV (*p* = 0.04). These findings are similar to our findings. However, other studies have shown that HGS in older adults is not associated with arterial stiffness (25). The results of the ANSAN cohort study in Korea showed that baPWV and CIMT were not correlated with HGS (36), which was partially consistent with our results. In addition, our study also confirmed that baPWV was negatively correlated with HGS, but CIMT was not correlated with HGS. This difference may be due to the fact that baPWV and CIMT reflect different vascular angles. BaPWV was used as a marker of arterial stiffness, reflecting the status of central and peripheral arterial stiffness (10, 13). However, CIMT is mainly an index of vascular morphology but can also reflect peripheral arterial stiffness to some extent (10, 37). When aging or disease occurs, atherosclerotic plaque deposition, vascular morphological changes, and CIMT changes, but vascular vasomotor function may not be affected at this time; moreover, vascular morphology is abnormal, but vasomotor function may be normal (38). Previous studies have shown that HGS is more closely related to central artery stiffness (36). This finding is consistent with our finding that baPWV is negatively correlated with HGS, while CIMT is not associated with HGS. Based on these findings, we conclude that arterial stiffness is associated with HGS.

With age, HGS may predict arterial stiffness (5, 6), and decreased HGS may lead to increased arterial stiffness (23). Arterial stiffness may be an intermediate factor in the association between HGS and cardiovascular events and mortality, which helps explain the association between HGS and cardiovascular disease and may be as effective as baPWV in predicting adverse cardiovascular outcomes (24, 36). Based on the results of these previous studies and the negative correlation between HGS and arterial stiffness found in our study, we propose a clinical method to improve arterial stiffness by enhancing HGS, thereby reducing the incidence of cardiovascular events. At a minimum, targeted intervention can improve vascular compliance after the diagnosis of low HGS. In screening prevention in the general population, HGS measurements are more accessible, faster, cheaper and more highly repeatable than baPWV measurements are. Moreover, studies have shown that improving HGS through exercise training is simple and effective (30, 31). Therefore, we encourage older adults to engage in active and effective physical exercise and improve HGS to reduce arterial stiffness and cardiovascular disease risk and improve their quality of life.

### 4.2 Association between arterial stiffness and low HGS

Our study revealed a significant relationship between arterial stiffness and loss of muscle strength. baPWV in the low HGS group was significantly higher than that in the normal HGS group (*p* < 0.001). With increasing per 1-SD in baPWV, the incidence of low HGS increased by 31.8% (OR = 1.318, *p* = 0.007). The odds ratio of low HGS in participants with arterial stiffness was 2.142 (*p* < 0.001). It also shows the significant effect of arterial stiffness on low HGS. BaPWV is an indicator of global arterial stiffness, a comprehensive measure of central and peripheral arterial stiffness, and the most commonly used indicator to study the relationship between muscle function and arterial stiffness (13). It is an independent risk factor for cardiovascular disease not only in people with hypertension but also in the general population (11). It has been suggested that central artery stiffness may mediate the association between HGS and cardiovascular events (36). HGS is associated with physical function and muscle mass (2, 39), and isometric HGS training can lower blood pressure and improve vascular function in hypertension (40). Research evidence for an association between increased arterial stiffness and low HGS is currently insufficient. Therefore, we focused on indicators of arterial stiffness to confirm that it is associated with low HGS. A study of an aging cohort divided PWV into two categories and observed that high PWV was associated with the risk of low HGS (OR = 6.12, *p* < 0.001), and low HGS went hand-in-hand with increased arterial stiffness (24). A Japanese study showed that a decrease in HGS may be associated with increased arterial stiffness in older adults in the community without significant cardiovascular disease (23). These are consistent with our findings. This study is the first to show that CCAID may be a risk-reducing factor for low HGS (OR per SD increase: 0.541, *p* < 0.001). A narrower CCAID had a higher chance of developing low HGS (*p* for trend<0.001). Interestingly, no relationship between CCAID and HGS was observed in the linear regression results of this study, while a correlation was observed in the multivariate logistic regression analysis, possibly due to the unknown nonlinear relationship between the two. CCAID is the minimum distance between the intimal cavity of the proximal and distal walls of the common carotid arterium (18). Carotid atherosclerosis is related to CCAID, and the prevalence differs among various communities. CIMT thickening may occur during carotid atherosclerosis, and CIMT is negatively correlated with CCAID, which may be accompanied by narrowing of CCAID (17, 18). This may lead to chronic low-level ischemia of muscle tissue, resulting in impaired muscle strength and decreased HGS, which may be why the narrower the CCAID is, the higher the risk of low HGS. Of course, more studies are needed to verify this result, and its complex mechanism needs to be further explored. Several possible explanations exist for the association between arterial stiffness and low HGS. First, vascular sclerosis leads to endothelial dysfunction, which causes dysregulation of blood flow and hypoperfusion of muscle tissue, resulting in chronic low-level ischemia (13). Second, inflammation may be a potential mechanism of arterial stiffness and low HGS. Studies have shown that inflammatory factors such as tumor necrosis factor and interleukin-6 are important factors that inhibit muscle synthesis and promote muscle atrophy, and inflammatory cytokines can cause changes in vascular dynamics, thus affecting muscle strength (21, 22). In addition, insulin resistance is a common pathway of decreased muscle strength and arterial stiffness, and an increase in advanced glycation end products causes vascular complications that may affect muscle strength (41, 42). However, in this study, neither multiple linear regression nor multivariate logistic regression analysis revealed any correlations of CIMT or the ABI with HGS (*p* > 0.05), which was similar to the results of previous studies (29, 36). This implied that changes in the ABI and CIMT may not affect HGS. More cross-sectional and longitudinal studies are needed to determine the relationship between these indicators. In conclusion, these results strengthen the hypothesis of a link between arterial stiffness and muscle strength.

### 4.3 Other associated factors of low HGS

In addition to indicators of arterial stiffness, we observed that age, BMI and Hb were associated with low HGS. Age was a factor associated with an increased risk of low HGS (OR per SD increase: 1.936, *p* < 0.001). Age-related muscle strength decreases significantly with age, and aging is strongly associated with low HGS, with HGS peaking in young and middle age and then showing a gradual downward trend (43). Age is an independent risk factor for low HGS (24, 44). BMI is the most common anthropometry dividing overweight and obesity, and a Japanese study noted that participants with low HGS had a lower BMI those with normal HGS, but were significantly associated with the occurrence of low HGS (OR = 0.71, 95% CI: 0.59–0.85, *p* < 0.001) (45). A cross-sectional study from China showed that BMI is a protective factor against low HGS and muscle mass in older adults (46). A prospective cohort study with 3 years of follow-up showed that a low BMI may be an indicator of frailty status and may lead to accelerated loss of HGS (47). These findings are consistent with the results of the present study. However, some studies have shown that overweight or obese people usually have insufficient exercise and excessive body fat. More body fat infiltrates muscles, making them more prone to low HGS (43). Therefore, a healthy lifestyle and diet and maintaining a normal BMI are also crucial for the muscular system. Hb is an important indicator of anemia. Low hemoglobin levels and anemia are common in older people and are robust markers of poor outcomes, such as frailty, decreased muscle strength, and reduced nutritional status (48). In this study, Hb was associated with the risk of low HGS (OR per SD increase: 0.795, *p* = 0.035). A cross-sectional and longitudinal study examining the association of Hb levels with low HGS, sarcopenia, and activities of daily living revealed that a 1 g/dL increase in Hb significantly reduced the risk of developing low HGS by 35% (OR = 0.75, *p* < 0.001, 48). Another Asian aging study confirmed a direct correlation between HGS and Hb level (49). Maintaining healthy Hb levels in older adults will be beneficial in maintaining nutritional muscle status, and measures can also be targeted from this perspective, which in turn will increase muscle strength.

## 5 Strengths and limitations

Our study has both strengths and limitations. To our knowledge, this is the first study to explore the association between arterial stiffness and HGS in relatively healthy older adults in Beijing, China. CCAID was revealed to be associated with low HGS for the first time. Moreover, this study combined baPWV, IMT, the ABI and CCAID as four arterial stiffness indicators and revealed a correlation between arterial stiffness and HGS after multi-indicator exploration. The association between vascular function and muscle health was established. We propose the clinical idea that improving HGS may improve arterial stiffness in older adults and further reduce the risk of cardiovascular events. However, this study has the following limitations. First, our cross-sectional study only found this phenomenon without intervention or long-term longitudinal follow-up to verify its accuracy. Second, we did not choose carotid-femoral pulse wave velocity (cfPWV), considered the gold standard for evaluating central arterial stiffness, as an indicator of arterial stiffness (10). However, baPWV is also widely used in many studies, and baPWV is highly correlated with cfPWV and is a recognized marker of arterial stiffness (11, 12). Finally, several factors, such as participant nutritional status (50, 51), sedentary lifestyle (52), physical activity (30), and depression (53), were found to be associated with HGS. However, these data were unavailable in this study, so we cannot explore the potential association between those factors and HGS.

## 6 Conclusion

In summary, we revealed that arterial stiffness is associated with HGS in relatively healthy older adults in Beijing, China. BaPWV, CCAID, age, BMI and Hb were associated with low HGS. The relationship between CCAID and low HGS was revealed for the first time. Higher baPWV and narrower CCAID increased the risk of low HGS. Our study has important implications to help establish the link between vascular function and muscle health in relatively healthy Chinese older adults. Improving HGS may improve arterial stiffness, thereby reducing cardiovascular risk and improving older adults’ quality of life.

## Data availability statement

The original contributions presented in the study are included in the article/Supplementary material. Further inquiries can be directed to the corresponding authors.

## Ethics statement

The studies involving humans were approved by the Ethics Committee of the General Hospital of the Chinese People’s Liberation Army. The studies were conducted in accordance with the local legislation and institutional requirements. The participants provided their written informed consent to participate in this study.

## Author contributions

YH: Data curation, Formal analysis, Investigation, Methodology, Writing – original draft, Writing – review & editing. YN: Data curation, Formal analysis, Investigation, Software, Writing – original draft. ZL: Investigation, Methodology, Writing – review & editing. RZ: Data curation, Formal analysis, Writing – review & editing. YC: Funding acquisition, Writing – review & editing. ZD: Investigation, Writing – review & editing. YZ: Funding acquisition, Writing – review & editing. QW: Investigation, Writing – review & editing. YW: Resources, Writing – review & editing. DZ: Investigation, Writing – review & editing. XS: Supervision, Writing – review & editing. GC: Supervision, Writing – review & editing. ZF: Funding acquisition, Supervision, Writing – review & editing. WZ: Conceptualization, Project administration, Supervision, Writing – review & editing. XC: Conceptualization, Funding acquisition, Project administration, Supervision, Writing – review & editing.
