# Supplementary material for: Arterial stiffness is associated with handgrip strength in relatively healthy Chinese older adults
Source: Front Nutr. 2024 Feb 9;11:1342411. doi: 10.3389/fnut.2024.1342411 (PMC10893589; doi:10.3389/fnut.2024.1342411)
Supplement: Supplementary file 1 [file Data_Sheet_1.docx]

***Supplementary Material***

**Supplementary Figures**

2217 subjects provided a questionnaire about basic information and past medical history

465 subjects were diagnosed with a disease on the exclusion list.

1752 subjects were required to undergo body measurements and laboratory testing

46 subjects failed to provide blood samples; 81 subjects had missing body measurements; 12 subjects had missing hand grip strength data; 62 subjects had missing brachial-ankle pulse wave velocity and ankle-brachial index data; 21subjects had missing common carotid artery internal diameter and carotid artery intima-media thickness data; 754 volunteers were under 60 years old.

776 subjects were finally included in the study

Figure S1. The flow diagram shows the screening and enrollment of the participants.


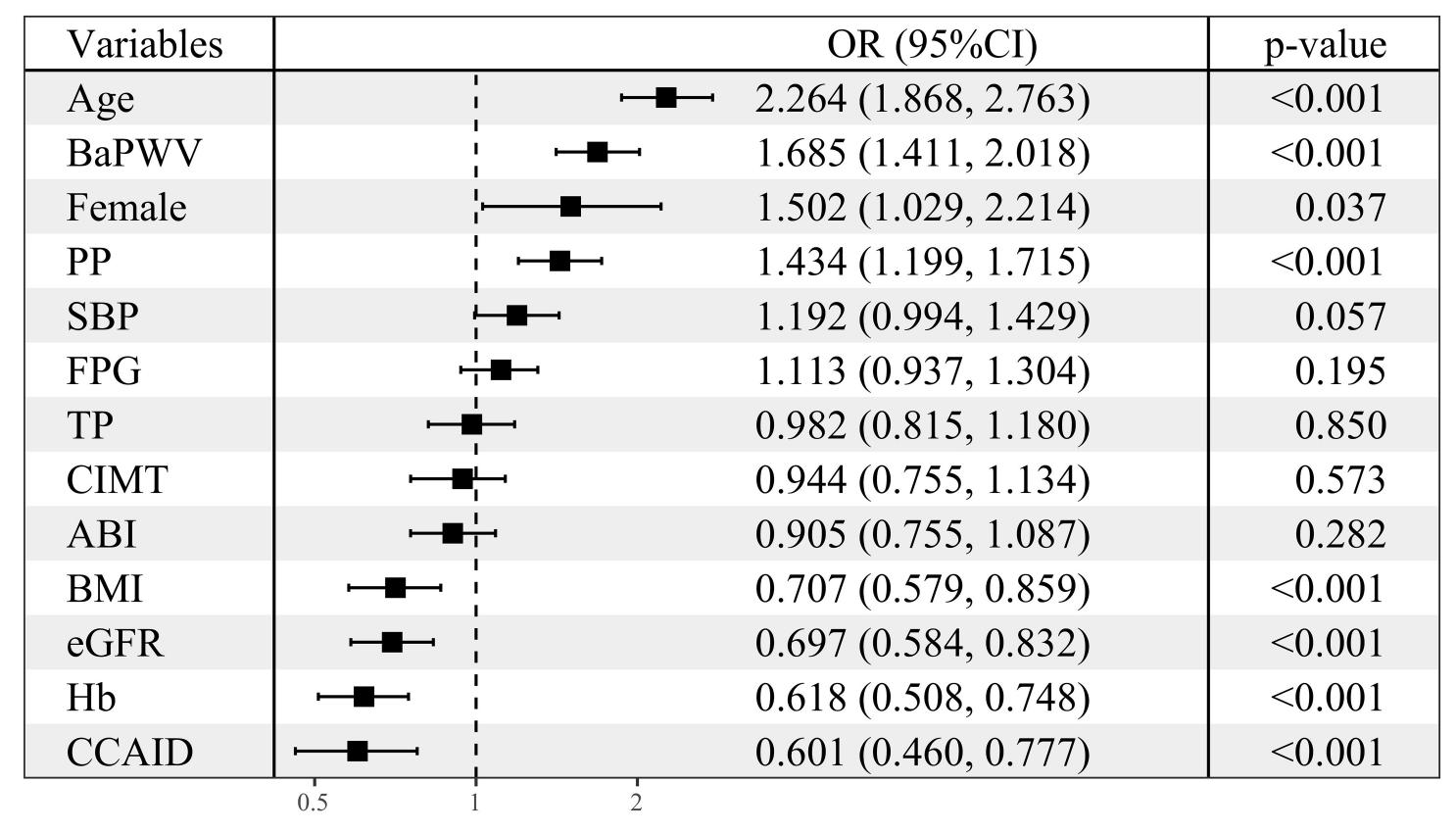


Figure S2 Univariate-Adjusted Standardized logistic regression analysis of low HGS

Abbreviations: HGS, handgrip strength; SBP, systolic blood pressure; PP, pulse pressure; baPWV, brachial-ankle pulse wave velocity; CIMT, carotid intima–media thickness; CCAID, common carotid artery internal diameter; ABI, ankle-brachial index; FPG, fasting plasma glucose; Hb, hemoglobin; TP, total protein; BMI, body mass index; eGFR, estimated glomerular filtration rate.
